# Long-Term Postnatal Follow-Up in Monochorionic TTTS Twin Pregnancies Treated with Fetoscopic Laser Surgery and Complicated by Right Ventricular Outflow Tract Anomalies

**DOI:** 10.3390/jcm12144734

**Published:** 2023-07-17

**Authors:** Stefano Faiola, Maria Mandalari, Chiara Coco, Daniela Casati, Arianna Laoreti, Savina Mannarino, Carla Corti, Dario Consonni, Irene Cetin, Mariano Lanna

**Affiliations:** 1 Fetal Therapy Unit ‘Umberto Nicolini’, Buzzi Children’s Hospital, 20154 Milan, Italy; 2 Department of Woman, Mother and Neonate, Buzzi Children’s Hospital, 20154 Milan, Italy; 3 Pediatric Cardiology Unit, Buzzi Children’s Hospital, 20154 Milan, Italy; 4 Epidemiology Unit, Fondazione IRCCS Ca’ Granda, Ospedale Maggiore Policlinico, 20122 Milan, Italy

**Keywords:** monochorionic twin, TTTS, fetoscopic laser surgery, prenatal RVOTA, CHD

## Abstract

Right ventricular outflow tract anomalies (RVOTAs), such as pulmonary stenosis (PS), pulmonary atresia (PA), and pulmonary insufficiency (PI), are typical cardiac anomalies in monochorionic twins, and they are complicated by twin-to-twin transfusion syndrome (TTTS). The aim of this study was to conduct a long-term postnatal cardiological evaluation of prenatal RVOTAs in monochorionic diamniotic twin pregnancies complicated by TTTS and treated with fetoscopic laser surgery (FLS) and to analyze possible prenatal predictors of congenital heart disease (CHD). Prenatal RVOTAs were retrospectively retrieved from all TTTS cases treated with FLS in our unit between 2009 and 2019. Twenty-eight prenatal cases of RVOTAs (16 PI, 10 PS, 2 PA) were observed out of 335 cases of TTTS. Four cases did not reach the postnatal period. CHD was present in 17 of the remaining 24 cases (70.8%), with 10 being severe (58.8%; 10/17); nine cases of PS required balloon valvuloplasty, and one case required biventricular non-compaction cardiomyopathy. The risk of major CHD increased with prenatal evidence of PS and decreased with the gestational age at the time of TTTS and with the prenatal normalization of blood flow across the pulmonary valve. Despite treatment with FLS, the majority of monochorionic diamniotic twin pregnancies complicated by TTTS with prenatal RVOTAs had CHD at long-term follow-up.

## 1. Introduction

Right ventricular outflow tract anomalies (RVOTAs), such as pulmonary stenosis (PS), pulmonary atresia (PA), and pulmonary insufficiency (PI), have been described as cardiac anomalies that are typical in monochorionic twin (MC) pregnancies complicated by twin-to-twin transfusion syndrome (TTTS), with a prevalence of 7–9% in the recipient twin (RT) in untreated pregnancies [1,2]. RT cardiomyopathy might be the consequence of the passage from donor to recipient of blood volumes and vasoactive peptides of the renin–angiotensin system through placental anastomosis; these increase vascular resistance, leading to higher pre- and afterloads on the left and right sides of RT hearts [3,4]. Fetoscopic laser surgery (FLS) of placental vascular anastomosis has been identified as the best treatment for TTTS [5]. Furthermore, previous studies demonstrated how FLS, which interrupts the passage of blood and vasoactive mediators, leads to an improvement in the right ventricular systolic and diastolic function of the RT [6]. However, in the ex-RT, the persistence or even the appearance of an RVOTA weeks after FLS or transient cardiac involvement in the ex-donor twin (DT) has been reported, despite successful FLS [7]. The primary aim of the present study was to evaluate long-term postnatal cardiological evaluations of cases with a prenatal RVOTA in a group of MC diamniotic (MC/DA) twin pregnancies complicated by TTTS and treated with FLS at a single center. The secondary aim was to analyze possible prenatal predictors of postnatal congenital heart disease (CHD).

## 2. Materials and Methods

This is a retrospective descriptive analysis of all MC/DA pregnancies complicated by TTTS and treated with FLS at the “Umberto Nicolini” Fetal Therapy Unit of the Vittore Buzzi Children’s Hospital in Milan (Italy) between January 2009 and January 2019. TTTS was defined according to the Eurofetus criteria (i.e., polyhydramnios of a ≥8 cm maximum vertical pocket in the recipient or ≥10 cm from 20 weeks of gestation onwards and oligohydramnios of a ≤2 cm maximum vertical pocket in the donor), and the Quintero Staging system was used to classify the severity of TTTS [8].

For each case, a detailed ultrasound anatomical evaluation including echocardiography was carried out for both MC/DA twins by using a GE Voluson 730, Expert, and GE E8, GE Healthcare, Zipf, Austria.

The presence, absence, and types of RVOTAs were noted. Prenatal RVOTAs were classified as pulmonary stenosis (PS) if a forward turbulent flow was detected across the pulmonary valve (PV) with aliasing and a peak systolic velocity (PSV) of >100 cm/sec, as pulmonary insufficiency (PI) if a bidirectional flow was identified across the PV, and as pulmonary atresia (PA) if no flow was detectable across the PV, with exclusive ductal reverse flow in the pulmonary artery [9]. At each ultrasound evaluation, in addition to the blood flow across the PV, the following parameters were recorded to assess the cardiovascular profile: the cardiothoracic circumference ratio (C/T) measured at a cross-sectional section through the fetal chest; tricuspid and mitral regurgitation, which was classified as mild if the jet was protosystolic, moderate if proto-mesosystolic, and moderate if holosystolic [7]; the ductus venosus a-wave flow, which was reported as present, absent, or reversed. The presence or absence of ascites was recorded. Pregnancies were also screened for selective fetal growth restriction (sFGR). sFGR was diagnosed based on an estimated fetal weight (EFW) of less than the 10th percentile in one twin and an intertwin EFW difference of >25%.

FLS was performed by using the selective technique until January 2012 and then with the Solomon technique from that date onwards [10].

Post-FLS fetal assessments were performed 24 and 48 h after the procedure, followed by prenatal echocardiographic assessments, which were performed at our center at 1 week and 1 month after the treatment, and all cardiovascular parameters were recorded. In addition, the patients who were directly followed in our hospital underwent a weekly assessment. All cases of MC/DA twin pregnancies with a prenatal diagnosis of an RVOTA were delivered at our hospital or at tertiary hospitals, allowing the newborns to undergo a formal cardiac assessment within 24 h of delivery. Postnatal examinations of patients delivered in our hospital were carried out by a dedicated pediatric cardiologist. The right outflow tract was evaluated and the transpulmonary mean and maximum gradients were recorded to assess the presence and severity of the abnormality [11]. The postnatal criterion for the presence of PS was an echocardiographic ventricular to pulmonary artery pressure gradient of >20 mmHg. In these patients, a different postnatal cardiological follow-up was scheduled according to the type and severity of the PS; they were monitored particularly closely during the first year of life due to the high risk of progression. After the first year of life, mild cases (peak gradient < 36 mmHg) were evaluated once or twice per year, while moderate cases (peak gradient: 36–64 mmHg; mean gradient: ≤50 mmHg) were assessed every 1–3 months. Patients with severe pulmonary stenosis (mean gradient: ≥50 mmHg) and pulmonary atresia underwent balloon valvuloplasty (BV) [12]. To ensure optimal follow-ups, in 2022, we contacted the mothers of all of the prenatal RVOTA cases and asked them to send us all pediatric cardiac evaluations, including their last clinical and echocardiographic report. Presence or absence of cardiac involvement and postnatal treatment (BV or medical therapy) was recorded under the supervision of our pediatric cardiologists. The CHD cases were divided into two categories: major if they required surgical or medical treatments or minor if they only required a clinical follow-up.

All women provided written informed consent for further clinical evaluation, and the study was approved by the ethics committee of Milan Area 1.

Survival analyses were performed by calculating the Kaplan–Meier failure functions and performing log-rank tests. Crude Cox models were used to calculate hazard ratios (HRs) and 95% confidence intervals (CIs). Analyses were performed with Stata 17 (StataCorp. 2021).

## 3. Results

During the study period, 28 prenatal cases of RVOTAs were observed in 335 MC/DA twin pregnancies complicated by TTTS and treated with FLS.

The prenatal data of these fetuses are shown in Table 1. As a type of prenatal RVOTA, PI was present in 16 cases (57.1%, 16/28), PS was present in 10 cases (35.7%, 10/28), and PA was present in 2 cases (7.1%, 2/28). The time at which RVOTAs appeared was related to TTTS and FLS; in three cases (10.7%), RVOTAs developed before TTTS; in 12 cases (42.8%), they developed at the same time as TTTS, and in 13 cases (46.4%), they developed after FLS. RVOTAs involved the RT in 24 cases (85.7%) and the DT in 4 cases (14.2%). All cases in the DTs developed after FLS; among them, there were three PIs and one PA, for an overall RVOTA incidence in DT of 1.5% (4 of the 258 DTs survived from FLS until birth).

Among the RTs, we observed 24 cases of RVOTAs. In three RTs, RVOTAs (two PSs and one PI) were observed before TTTS developed, and in 12 RTs, RVOTAs were observed at the time of the TTTS diagnosis: eight PIs, one PAs, and three PSs. Overall, in 4.5% (15/335) of the RTs, prenatal RVOTAs were present at the time of the TTTS diagnosis.

In nine RTs, RVOTAs were observed only after FLS, with an incidence in this subgroup of 3.5% (9 of the 259 RTs who did not exhibit an RVOTA before FLS and survived until birth). In this subgroup, we recorded four PIs and five PSs and, in four cases (three PIs and one PS), recurrent TTTS was also present.

In our population, the total incidence of prenatal RVOTAs was 8% (4.5% before FLS and 3.5% after FLS).

Out of the 28 prenatal RVOTA cases, we had two cases of fetal death. One death occurred after FLS, and there was one termination of the pregnancy due to the critical condition of both twins (an ex-recipient with hydrops and an ex-donor with sFGR and reversed flow in the umbilical arteries).

**Table 1:** Prenatal outcomes of twins with RVOTAs in MC pregnancies with TTTS.

Case N	RVOTA		Twin with RVOTA	Onset RVOTA	Severe TV-R	Severe MV-R	DV a-Wave	C/T Ratio ≥ 0.55	TTTS		Additional US Findings Detected during Pregnancy	Last US (Weeks)	Normalization FVW-PV	Pregnancy Outcome
	Type	GA (Weeks)							Type	GA (Weeks)				
**1**	PS	15.0	R	Before TTTS	yes	yes	no	yes	3	15.6	IUD D 24 h after FLS. Biventricular hypertrophy	21.6	no	Alive
**2**	PS	20.3	R	After FLS	yes	no	yes	yes	3	18.2	None	35.2	no	Alive
**3**	PS	19.6	R	Before TTTS	no	no	yes	no	1	21.4	None	26.0	no	Alive
**4**	PI	20.6	R	At the time of TTTS	yes	no	no	yes	4	20.6	PA at 23 weeks	24.1	no	IUD
**5**	PA	21.0	R	At the time of TTTS	yes	no	no	yes	4	21.0	Dilated cardiomyopathy	NA	yes	Alive
**6**	PI	21.1	D	After FLS	yes	no	no	no	2	20.0	Ex-donor tricuspid valve dysplasia 11 weeks after FLS	31.1	yes	Alive
**7**	PS	23.3	R	After FLS	no	no	yes	no	2	17.6	PS 5 weeks after FLS	23.3	no	Alive
**8**	PI	21.3	R	At the time of TTTS	yes	yes	no	yes	4	21.3	None	27.0	yes	Alive
**9**	PS	17.3	R	At the time of TTTS	yes	yes	no	yes	3	17.3	IUD donor 24 h after FLS.	30.3	no	Alive
**10**	PI	23.5	D	After FLS	yes	no	no	yes	2	22.5	Ex-donor hydrops due to heart failure 6 days after FLS	33.1	yes	Alive
**11**	PS	23.0	R	At the time of TTTS	yes	yes	no	yes	4	23.0	Myocardial hypertrophy	35.3	no	Alive
**12**	PA	23.6	D	After FLS	no	no	yes	no	2	21.5	Ex-donor: hydrops due to heart failure 13 days after FLS; therapy with Digoxin from 26 weeks. Mirror syndrome	30.0	yes	Alive
**13**	PS	24.0	R	After FLS	yes	no	yes	yes	1	20.1	TTTS recurrence 3 weeks after FLS; amniodecompression	33.0	yes	Alive
**14**	PS	18.2	R	After FLS	yes	no	yes	yes	2	16.3	Ex-donor IUD 14 days after FLS	31.0	yes	Alive
**15**	PI	22.4	D	After FLS	no	no	no	yes	2	19.6	Hydrops due to heart failure 12 days after FLS, spontaneously resolved	34.4	yes	Alive
**16**	PS	17.4	R	At the time of TTTS	yes	no	yes	yes	2	17.4	Ex-donor sFGR with AEDF	30.1	no	Alive
**17**	PS	26.2	R	After FLS	no	yes	yes	yes	3	21.0	IUD ex-donor sFGR with REDF. Myocardial hypertrophy	27.3	yes	Alive
**18**	PI	26.3	R	Before TTTS	no	no	no	yes	3	26.4	IUD ex-donor 14 days after FLS	34.1	yes	Alive
**19**	PI	20.0	R	After FLS	yes	yes	no	yes	2	19.0	TTTS persistence after FLS; 2° FLS after 7 days. Heart failure in ex-recipient with hydrops; ex-donor with sFGR	22.1	no	TOP (recurrence of TTTS with hydrops of the recipient and donor severe sFGR with REDF in UA)
**20**	PI	24.4	R	At the time of TTTS	yes	no	no	yes	4	24.4	None	26.0	yes	Alive
**21**	PI	19.5	R	After FLS	yes	yes	no	yes	1	18.5	Ascites 24 h after FLS	34.5	yes	Alive
**22**	PI	17.4	R	At the time of TTTS	yes	no	no	yes	4	17.4	Myocardial hypertrophy	31.1	yes	Alive
**23**	PI	25.1	R	At the time of TTTS	yes	no	no	yes	4	25.1	MRI before FLS: IVH grade 1	30.0	yes	Alive
**24**	PI	22.2	R	At the time of TTTS	yes	no	no	yes	4	22.2	Myocardial hypertrophy	25.3	yes	Alive
**25**	PI	22.6	R	After FLS	yes	no	no	yes	2	22.0	Ex-donor with SNC damage. Selective TOP of ex-donor. Amniodecompression 2 days after FLS. Myocardial hypertrophy.	23.2	yes	Alive
**26**	PI	20.4	R	At the time of TTTS	yes	yes	no	yes	3	20.4	TAPS sequence 48 h after FSL. Myocardial hypertrophy	22.6	no	Alive
**27**	PI	26.3	R	After FLS	no	no	no	no	1	20.1	TTTS recurrence 6 weeks after FLS; 3 amniodecompression; heart failure of ex-recipient treated with Digoxin from 26 weeks. Biventricular hypokinesia	28.5	no	Alive
**28**	PI	23.4	R	At the time of TTTS	yes	yes	no	yes	4	23.4	Ex-recipient heart failure from 26 weeks treated with Digoxin; ex-donor sFGR. Myocardial hypertrophy	33.0	yes	Alive

**Figure 1:**
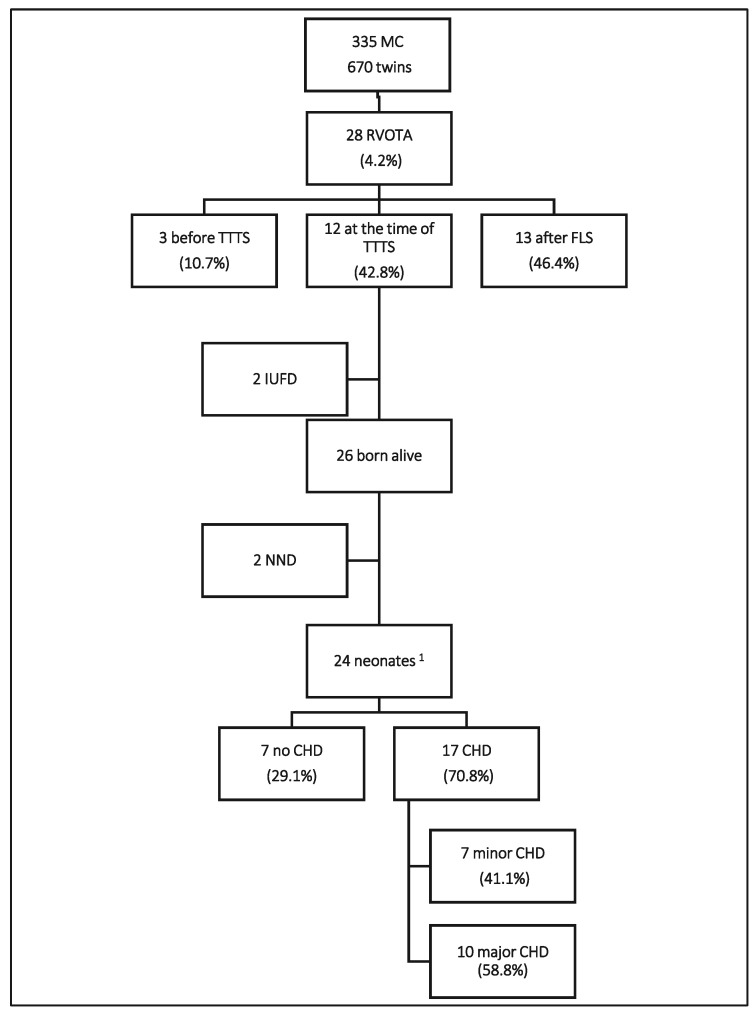
Study population. MC: monochorionic pregnancy; RVOTA: right ventricle outflow tract abnormality; TTTS: twin–twin transfusion syndrome; IUFD: intrauterine fetal demise; NND: neonatal death; CHD: congenital heart defects. ^1^ Study population.

RVOTA: right ventricle outflow tract abnormality; MC: monochorionic pregnancy; US: ultrasound; GA: gestational age; TV-R: tricuspid valve regurgitation; MV-R: mitral valve regurgitation; C/T: cardiothoracic ratio; sFGR: selective fetal growth restriction; D: donor; R: receiving; pPROM: premature preterm rupture of membranes; IVH: intraventricular hemorrhage; TAPS: twin anemia polycytemia sequence; TTTS: twin–twin transfusion syndrome; PS: pulmonary stenosis; PI: pulmonary insufficiency; PSI: pulmonary steno-insufficiency; PA: pulmonary atresia; FLS: fetoscopic laser surgery; REDF in UA: reverse-end diastolic flow in umbilical artery; TOP: termination of pregnancy; IUD: intrauterine death. The postnatal data of the remaining 26 fetuses are shown in Table 2. In the postnatal population, we recorded two neonatal deaths: one due to prematurity and the other due to heart failure. Overall, among these 28 prenatal RVOTA cases, 24 survived the perinatal period and formed our study population (Figure 1).

We were able to collect the postnatal cardiac long-term follow-up data of all 24 patients who survived the perinatal period, for a median of 9.5 years (range 3–13 years). In 17 patients (70.8%; 17/24), a CHD was present, with a major CHD in 10 cases (58.8%; 10/17); there were nine cases with severe PS requiring BV and one with biventricular non-compaction cardiomyopathy. In the other seven cases (41.1%; 7/17), the long-term FU showed a minor CHD in the form of dysplastic AV valves in five cases, left ventricular hypertrabeculation in one case, and mild pulmonary steno-insufficiency in another case.

The prognostic role of prenatal parameters in major CHDs is shown in Table 3. The risk of a major CHD was almost five times higher in the case of prenatal PS than in the case of PI (HR 4.71, 95° CI: 1.18–18.7). No major CHDs were observed if the DT presented prenatal RVOTAs (P: 0.07). The risk of major CHDs decreased with the gestational age at the time of TTTS (HR 0.69, 95° CI: 0.49–0.96). The prenatal normalization of the blood flow across the pulmonary valve was associated with a strongly reduced risk (HR 0.09, 95° CI: 0.02–0.42). No significant associations were observed between major CHDs at the long-term follow-up and the following prenatal parameters: the onset of an RVOTA (before or at the time of TTTS or after FLS), the TTTS stage, the presence of sFGR, C/T > 0.55, abnormal DV, severe tricuspid valve regurgitation, severe mitral valve regurgitation, gestational age, and weight at delivery.

## 4. Discussion

To the best of our knowledge, this is the first study to analyze long-term postnatal cardiological evaluations of cases with prenatal RVOTAs in a homogeneous group of MC/DA twin pregnancies complicated by TTTS and treated with FLS. Furthermore, in our series, we checked for RVOTAs in the DT as well, finding an RVOTA incidence in DTs of 1.5%. The RVOTA incidence in DTs has never been investigated before in studies of TTTS. Alterations in the cardiac function of DTs after laser coagulation are common and were previously described by Van Mieghem et al. [13]. They arise due to a relative overload occurring in the DT after FLS in the form of the vasoactive peptides and blood volume previously given to the RT, interrupting the passage through the placental anastomoses, and they must be managed by the DT themselves. However, in four of our DTs, these alterations had a significant impact on cardiac function, causing hydrops in the prenatal period and minor CHDs, such as AV dysplasia, at the long-term follow-up. The incidence of RVOTAs in the RTs in our series (8%) was similar to the value of 7.5% reported by Chang et al. [14], even though PI, as a type of RVOTA, was not considered in that study.

The onset of RVOTAs before TTTS has not previously been described. However, we recently published cases of RVOTAs in MC pregnancies without TTTS, especially in cases of sFGR with amniotic fluid discrepancies [15,16]. A possible pathophysiologic explanation for these findings is that the uneven and mismatched transfer of blood volume and vasoactive peptides is enough to cause cardiac adaptation without TTTS typically being found; this may develop afterwards, as in the three cases described in the present study.

The onset of RVOTAs, especially PS, several weeks after FLS without signs of recurrence was already described in a prospective TTTS series by Eschbach et al., where it was correlated with mild postnatal PS. In our series, in two cases of PS that developed two and five weeks after FLS, severe postnatal PS was observed, requiring BV. Those cases may demonstrate that, even in the second trimester, the processes of valve maturation are still taking place and valvular damage can appear weeks after the adverse event.

Regarding the types of RVOTAs, we found a strong correlation between prenatal PS and the risk of major CHDs, with 70% of prenatal PS cases requiring BV for severe postnatal PS. This finding could be explained by prenatal PS being the expression of an organic valve pathology rather than a functional alteration. Therefore, it is not cured by the improvement of the right ventricular systolic and diastolic function that is visible after the FLS. Furthermore, our finding of a strong risk reduction for major CHDs with the prenatal normalization of blood flow across the pulmonary valve (PV) could be the expression of a prenatal RVOTA as a transient functional abnormality rather than an organic disease. We also found that all patients with a postnatal dysplastic AV valve exhibited a normalization of blood across the PV before birth and never developed PS. We could not find an explanation for this finding. In addition, we found an association between the gestational age at the time of TTTS and a risk of major CHDs, with a risk reduction of 31% for each additional gestational week. This finding is in agreement with those of other studies [14,17] and confirms how alterations in cardiac hemodynamics in the early stages of embryogenesis lead to irreversible organic changes. However, treatment with FLS can ensure the regression of functional RVOTAs before they become organic. This is thoroughly demonstrated by the finding in our recent publication that RVOTA incidence at birth in RTs was halved in the FLS group compared with a TTTS group that was treated before 2004 with amnioreduction (4.5% vs. 9.4%, respectively) [18].

Biventricular non-compaction cardiomyopathy is a rare cardiac abnormality that is characterized by a two-layered myocardium, numerous prominent trabeculations, and deep intertrabecular recesses communicating with the ventricular cavity [19]. To the best of our knowledge, this is the first ventricular non-compaction cardiomyopathy (VNC) to be reported in an MC pregnancy.

VNC is a genetic cardiomyopathy with a multifactorial origin involving mutations of the genes that encode the sarcomeric, cytoskeletal, and nuclear membrane proteins [20]. In our case series, this abnormality was present as a discordant abnormality in an MC recipient twin who exhibited severe biventricular dysfunction as a fetus. This finding points to an underlying epigenetic origin that could act by modifying one of the multiple pathways involved in normal myocardial compaction [21].

Strengths and limitations: This study has many strengths. One major strength is the homogeneity of the population of MC/DA twin pregnancies complicated by TTTS and treated with FLS. Another strength is that all prenatal evaluations were conducted in a third-level center with extensive experience with MC twins. Moreover, a complete postnatal follow-up was conducted in all cases.

The main limitations of this study are its retrospective nature and the lack of centralization or a shared protocol in the postnatal follow-up examinations, as many of these patients were not born in our hospital.

A multicenter prospective study on prenatal RVOTAs in MC/DA twin pregnancies complicated by TTTS and treated with FLS is needed to better understand their incidence, prenatal evolution, and long-term postnatal cardiological outcomes.

## 5. Conclusions

Our study demonstrates that following fetoscopic laser surgery, the majority of babies born of monochorionic pregnancies complicated by TTTS with a prenatal right ventricular outflow tract anomaly develop a congenital heart disease according to long-term follow-up observations. TTTS cases complicated by right ventricular outflow tract anomalies should be referred to a tertiary care hospital where specialized prenatal and postnatal cardiac evaluations, treatments, and long-term follow-ups are available.

## Figures and Tables

**Table 2:** Postnatal outcomes of twins with RVOTAs in MC pregnancies with TTTS.

Case N *	GA at the Delivery	BW (gr)	Neonatal Type of RVOTA	Years of Follow-Up	CHD at Follow-Up
**1**	32	1850	PS	4	Major CHD: severe PS with BV
**2**	35	2960	PS	8	Major CHD: severe PS with BV and closure of the ductus arteriosus
**3**	33	1500	PA	7	Major CHD: PA with intact ventricular septum, BV, iatrogenic hemopericardium during the procedure. Persistence of mild PI; possibility of re-intervention
**5**	36	1960	0	8	Minor CHD: left ventricular hypertrabeculation
**6**	34	1490	0	9	Minor CHD: tricuspid dysplasia with mild to moderate TV-R
**7**	34	2020	PS	5	Major CHD: severe PS with BV. Residual PSI at FU
**8**	34	1690	0	6	None
**9**	37	2680	PS	11	Major CHD: severe PS with BV
**10**	33	1793	0	10	None
**11**	35	2170	PS	11	Major CHD: severe PS with BV, atrial septal defect closure. Planned replacement of the PV due to the residual severe PI
**12**	30	1200	0	11	Minor CHD: neonatal correction of patent ductus arteriosus; dysplastic atrioventricular valves; persistence of moderate TV-R and MV-R at the follow-up, without the need for surgical correction
**13**	33	2150	0	10	Minor CHD: tricuspid dysplasia with moderate TV-R
**14**	38	2700	PS	10	Minor CHD: mild PSI, gradient 34 mmHg. Stationary follow-up
**15**	35	2340	0	12	None
**16**	31	1630	PS	10	Major CHD: severe PS with BV; hypertrophic cardiomyopathy
**17**	28	1430	NA	11	None
**18**	35	2285	0	3	None
**20**	26	1100	NA	NA	NND due to severe prematurity at birth, severe RDS and heart failure with biventricular dilatation and systolic dysfunction
**21**	34	2660	0	10	None
**22**	32	1700	PS	5	Major CHD: severe PS with BV
**23**	33	1800	0	6	None
**24**	26	1000	PI	4	Minor CHD: mitral dysplasia with MV-R: no intervention
**25**	24	500	PS	13	Major CHD: severe PS with BV
**26**	28	690	0	11	Major CHD: cardiomyopathy at 7 years, non-compact myocardium
**27**	28	990	NA	NA	NND due to heart failure
**28**	33	1900	0	9	Minor CHD: tricuspid and mitral dysplasia with moderate TV-R and mild MV-R at birth

RVOTA: right ventricle outflow tract abnormality; MC: monochorionic pregnancy; TTTS: twin–twin transfusion syndrome; BW: birth weight; CHD: congenital heart defect; PS: pulmonary stenosis; PI: pulmonary insufficiency; PSI: pulmonary steno-insufficiency; PA: pulmonary atresia; TV-R: tricuspid valve regurgitation; MV-R: mitral valve regurgitation; BV: pulmonary balloon valvuloplasty; ND: neonatal death; * Cases 4 and 19 are not shown as intrauterine deaths.

**Table 3:** Prenatal variables in 24 children with RVOTAs.

Variable	N or Median (Range)	Cases with Major CHDs N (%) or Median (Range)	Hazard Ratio (95%CI)
Pulmonary insufficiency	12	3 (25)	1.00 (Reference)
Pulmonary stenosis	10	7 (70)	**4.71 (1.18–18.7)**
Pulmonary atresia	2	0	NC
Recipient twin with an RVOTA	20	10 (50)	1.00 (Reference)
Donor twin with an RVOTA	4	**0**	NC
RVOTA at the time of TTTS	10	5 (50)	1.00 (Reference)
RVOTA before TTTS	3	2 (67)	1.48 (0.28–7.69)
RVOTA developed after FLS	11	3 (27)	0.52 (0.12–2.19)
Gestational age at time of TTTS (weeks)	21.2 (16.4–23.6)	18.1 (15.9–23)	**0.69 (0.50–0.97)**
TTTS Stage 1–2	12	5 (42)	1.00 (Reference)
TTTS Stage 3–4	12	5 (42)	1.28 (0.37–4.43)
No selective fetal growth restriction	12	5 (42)	1.00 (Reference)
Selective fetal growth restriction	12	5 (42)	1.16 (0.33–4.04)
Cardio/thoracic ratio < 0.55	8	3 (37)	1.00 (Reference)
Cardio/thoracic ratio ≥ 0.55	16	7 (43)	1.49 (0.38–5.86)
No reversed a-wave in ductus venosus	8	3 (37)	1.00 (Reference)
Reversed a-wave in ductus venosus	16	7 (43)	1.25 (0.32–4.85)
No severe insufficiency in TV	7	2 (29)	1.00 (Reference)
Severe insufficiency in TV	17	8 (47)	1.62 (0.34–7.79)
No severe insufficiency in MV	13	4 (31)	1.00 (Reference)
Severe insufficiency in MV	11	6 (54)	2.23 (0.63–7.93)
No prenatal normalization of the flow across the pulmonary valve	8	8 (100)	1.00 (Reference)
Prenatal normalization of the flow across the pulmonary valve	16	2 (12)	**0.09 (0.02–0.42)**
Gestational age at delivery (weeks)	33 (24–38)	32 (24–35)	0.89 (0.73–1.08)
Weight at delivery (gr)	1.850 (500–2.960)	1.770 (500–2170)	0.99 (0.99–1.00)

N: numbers; RVOTAs: right ventricular outflow tract anomalies; FLS: fetoscopic laser surgery; TV: tricuspid valve; MV: mitral valve; CI: confidence interval; NC: not calculable.

## Data Availability

The data that support the findings of this study are available on request from the corresponding author.
